# Knowledge about blood donation in patients of a hospital in Amazonas, Peru

**DOI:** 10.17843/rpmesp.2022.392.10829

**Published:** 2022-06-30

**Authors:** Nelson Santisteban, Jorge Osada

**Affiliations:** 1 Hospital El Buen Samaritano, Seguro Social de Salud, Amazonas, Peru. Hospital El Buen Samaritano Seguro Social de Salud Amazonas Peru; 2 Instituto de Evaluación de Tecnologías en Salud e Investigación, Seguro Social de Salud, Lima, Peru. Instituto de Evaluación de Tecnologías en Salud e Investigación Seguro Social de Salud Lima Peru

**Keywords:** Blood Donors, Knowledge, Outpatient Clinics, Hospital (source: MeSH NLM).

## Abstract

This study aimed to determine the level of knowledge about blood donation among outpatients from a hospital in Bagua Grande. A descriptive cross-sectional study was conducted from May to July 2019. We developed and applied a structured questionnaire. A total of 244 outpatients were recruited through systematic sampling. Participants obtained a mean number of correct answers of 8.90 (SD: 3.25) points out of 21. Age and educational level were found to be correlated with the score (rho = -0.21; p < 0.001 and rho = 0.38; p < 0.001, respectively). Place of origin and occupation affected the score (p < 0.001). We found a low level of knowledge in the studied population, but this would not be related to previous history of donation. Interventions to improve blood donation should not only focus on providing knowledge, since other factors would be more related to this result.

## INTRODUCTION

Blood donation saves lives worldwide; however, it is not frequent in the Americas and has a low percentage. The Pan American Health Organization (PAHO) and various health organizations in the region encourage voluntary blood donation. In Peru, the National Program of Hemotherapy and Blood Banks (PRONAHEBAS) has made great efforts to increase voluntary blood donations in the population, but unfortunately the effects have not been significant ^1^.

Attitudes, beliefs, and knowledge associated with blood donation can affect donor recruitment in the population. Blood donation is influenced by factors such as education and cultural context ^2^. Lack of knowledge and misconceptions about donation can contribute to a lack of initiative for voluntary donation ^3^.

Several authors agree that the population has general knowledge about blood, transfusion and donation, but little knowledge about specific aspects ^4^ ^-^ ^9^. This lack of information could prevent them from making an informed decision about this process. In addition, the presence of taboos and myths about blood donation are common and have a negative influence ^10^. These barriers include fear of blood extraction, disease transmission, commercialization of blood, religion, loss of fertility or sexual performance ^2^, lack of need for donors, weakness or anemia ^10^.

Studies conducted in Peru coincide with previous research, but they focus predominantly on urban populations ^11^ or those related to the health sciences ^12^ ^,^ ^13^, and most of them are published as gray literature. Although these studies provide interesting data, their results cannot be extrapolated to the national reality, because it is made up of heterogeneous cultural contexts with a diversity of languages, dialects and traditions that could influence the knowledge, attitudes and experiences of people about blood donation. Bagua Grande is a city located in the Peruvian jungle with great commercial activity, and it concentrates people from different areas of the jungle, therefore, it allows the evaluation of diverse regional traditions and beliefs.

This study aimed to determine the level of knowledge about blood donation among patients from the Hospital El Buen Samaritano de Bagua Grande. We also explored whether this level of knowledge was related to the history of blood donation. Identifying the level of knowledge will allow designing specific interventions to improve the perception of blood donation and its frequency.

**KEY MESSAGES**

Motivation for the study: The frequency of blood donation is low; therefore, it is necessary to understand this problem in order to improve access to donation.

Main findings: The level of knowledge found in the population of Bagua Grande is low and does not seem to be related to the history of donation.

Implications for public health: Interventions to improve blood donation should not only focus on providing knowledge, since other factors would be more related to this finding.

## THE STUDY

Cross-sectional descriptive study carried out in a first level hospital of the Peruvian social security located in the city of Bagua Grande in the Amazonas region in Peru, from May to July 2019. In order to define the sample, the total population was considered as the people who attended the hospital’s outpatient services during the second quarter of 2019. Therefore, the total population was defined as 667 people, according to statistical data provided by the same health center. By considering an estimated proportion of 50% for sample calculation, we obtained a sample of 244 people. Likewise, we applied systematic random sampling, with a skip interval (k) of two persons, to all those in the outpatient services of the hospital who passed through the only access to that area.

We prepared a structured questionnaire (supplementary material) based on the Technical Guide for the Selection of Human Blood and Hemocomponents Donors, used in Peru ^14^ and data from previous studies ^15^.

The questionnaire was divided into two sections; the first included characteristics of the respondents (age, sex, marital status, occupation, place of origin, religion, level of education and history of blood donation), and the second was focused on measuring knowledge. This last section was included 21 questions about blood, donation and its associated processes. All questions were closed-ended and multiple choice, with the exception of age in years. The questions regarding knowledge had only one correct answer which was considered as one point. The instrument was validated by the judgment of experts who were professional laboratory specialists with experience and postgraduate training in the area. The degree of agreement among the experts was determined with the Fleiss Kappa coefficient. The reliability of the instrument’s items was evaluated by means of a pilot test on 60 people who attended another hospital in the city, applying the Kuder Richardson coefficient, which showed a high value (0.77). Although there are instruments applied in other contexts, we decided to create a new instrument to adjust the knowledge to the national technical guide.

People over 18 years of age who attended the outpatient services of the hospital and who had a scheduled appointment at outpatient or diagnostic assistance services were invited to participate in the survey. By means of an oral pre-screening, we excluded those who had diseases that prevented them from adequately answering the questions. Participation was requested by informed consent. The survey was carried out from Monday to Saturday by the researchers, during the working hours of the health facility.

We carried out a descriptive analysis focused on presenting the frequency of knowledge globally and according to the sociodemographic data collected. We explored whether the evaluated variables were associated with the history of donation by means of a bivariate analysis according to the independent variables chosen and a robust Poisson multivariate analysis including variables with a p value < 0.20 in the bivariate analysis. Data analysis was carried out using the Stata v15 statistical package.

The study was approved by the Research and Ethics Committee of the Hospital Nacional Almanzor Aguinaga Asenjo of the Red Asistencial Lambayeque del Seguro Social de Salud (NIT: 4850-2019-684). Participation was voluntary, and informed consent was obtained from all those contacted. Questionnaires were stored separately from the informed consent forms to guarantee the anonymity of the participants during data processing.

## FINDINGS

We evaluated the total number of planned participants, that is, 244 individuals, who were between 19 and 71 years of age, most of them were women, had a university degree, had compulsory insurance, were employed, lived in an urban area and were Catholics. Likewise, most of the participants denied any history of having donated blood. The characteristics of the participants are shown in Table 1.

**Table 1:** Mean knowledge score according to participant characteristics.

Characteristics	n (244)	%	Mean score	p-value ^a^
Age ^b^	44.7	11.8	-0.2	<0.001 ^c^
Sex				0.827
Women	159	65.2	8.9±3.1	
Men	85	34.8	8.8±3.5	
Marital status				0.132 ^d^
Single	83	34.0	9.2±3.3	
Married	81	33.2	8.2±3.4	
Cohabitant	75	30.7	9.2±3.0	
Divorced	3	1.2	11.7±2.3	
Widower	2	0.8	10.0±2.8	
Type of insurance				<0.001 ^d^
Beneficiaries	60	24.6	7.5±3.3	
Compulsory	166	68.0	9.6±2.8	
Pensioner	2	0.8	14.5±0.7	
Agricultural	16	6.6	5.9±3.3	
Occupation				<0.001^d^
Employed	163	66.8	9.8±2.8	
Farmer	18	7.4	5.9±3.7	
Housewife	49	20.1	6.8±2.9	
Student	2	0.8	12.5±5.0	
Merchant	1	0.4	6	
Independent	8	3.3	8.3±2.4	
Retiree	2	0.8	12.0±2.8	
Unemployed	1	0.4	11	
Place of origin				<0.001
Urban	188	77.1	9.3±3.0	
Rural	56	23.0	7.4±3.6	
Religion				0.698 ^d^
Catholic	190	77.9	8.9±3.3	
Adventist	27	11.1	10.0±2.7	
Evangelic	1	0.4	4	
Israel of Jehovah	1	0.4	9	
Mormon	1	0.4	9	
World Missionary Movement (WMM)	2	0.8	8.0±1.4	
Nazarene	11	4.5	8.0±3.3	
Pentecostal	4	1.6	8.3±1.7	
Peregrine	3	1.2	7.7±4.2	
Jehovah witness	4	1.6	7.5±4.8	
Educational level				<0.001^d^
Primary school	21	8.6	5.1±3.5	
Secondary school	59	24.1	7.3±2.7	
Non-university higher education	74	30.3	10.1±2.8	
University higher education	90	36.9	9.8±2.7	
Donation				0.096
No	194	79.5	8.7±3.3	
yes	50	20.5	9.6±2.8	

a Student’s T

b Mean and standard deviation

c Spearman's Rho

d Kruskal Wallis. A nonparametric test was used because of the small number of some groups.

The mean of the participants’ correct answers was 8.90 (SD: 3.25) points, out of a possible total of 21. The percentage of positive answers for each question can be seen in Table 2. We explored whether the characteristics of the participants affected this score, observing that age was weakly correlated with the score (rho = -0.21; p < 0.001), as well as with the level of education (rho = 0.38; p < 0.001). We found a higher level of knowledge in people with compulsory insurance and pensioners (retirees) than in beneficiaries (workers’ relatives) and people with agricultural insurance; this was also observed when comparing employees with housewives and farmers, and when comparing people with higher education with people with primary or secondary education. Likewise, there was a non-significant tendency for people with a history of donation to have greater knowledge (Table 1).

**Table 2:** Characteristics of participants according to history of donation.

Characteristics	History of donation				p-value
	Yes		No		
	n (50)	%	n (194)	%	
Age ^a^	45.5	10.7	44.48	89.3	0.573^ b^
Sex					0.007
Women	24	15.1	135	84.9	
Men	26	30.6	59	69.4	
Marital status					0.116
Single	16	19.3	67	80.7	
Married	11	13.6	70	86.4	
Cohabitant	22	29.3	53	70.7	
Divorced	1	33.3	2	66.7	
Widower	0	0.0	2	100.0	
Insurance type					0.622
Beneficiary	9	15.0	51	85.0	
Compulsory	38	22.9	128	77.1	
Pensioner	0	0.0	2	100.0	
Agricultural	3	18.8	13	81.2	
Occupation					0.135
Employed	38	23.3	125	76.7	
Farmer	4	22.2	14	77.8	
Housewife	4	8.2	45	91.8	
Student	1	50.0	1	50.0	
Merchant	0	0.0	1	100.0	
Independent	3	37.5	5	62.5	
Retiree	0	0.0	2	100.0	
Unemployed	0	0.0	1	100.0	
Place of origin					0.707
Urban	40	21.3	148	78.7	
Rural	10	17.9	46	82.1	
Religion					0.249
Catholic	39	20.5	151	79.5	
Adventist	5	18.5	22	81.5	
Evangelic	0	0.0	1	100.0	
Israel of Jehovah	1	100.0	0	0.0	
Mormon	0	0.0	1	100.0	
World Missionary Movement (WMM)	1	50.0	1	50.0	
Nazarene	1	9.1	10	90.9	
Pentecostal	1	25.0	3	75.0	
Peregrine	2	66.7	1	33.3	
Jehovah’s Witness	0	0.0	4	100.0	
Education level					0.700
Primary school	3	14.3	18	85.7	
Secondary school	10	17.0	49	83.1	
Non-university higher education	18	24.3	56	75.7	
University higher education	19	21.1	71	78.9	

a Mean and standard deviation

b Student’s t

We explored whether the characteristics of the participants were related to the history of blood donation. Sex was significantly related to this event, as greater history of donation was found in men. No association was observed with the rest of the variables (Table 2). When exploring the relationship between the frequency of correct answers with the knowledge questions and the history of blood donation, we only found a relationship with question 2 (what is blood for?) (Table 3). A multivariate analysis was carried out, but we didn’t found differences with the bivariate analysis. Likewise, the subgroups for some variables were too small for an acceptable analysis (data not shown).

**Table 3:** Knowledge of participants according to the history of donation.

Knowledge (correct answers)	History of donation						p-value^ a^
	Yes		No		Total		
	n (50)	%	n (194)	%	n (244)	%	
Where is the blood formed?	14	25.9	40	74.1	54	22.1	0.258
What is blood for?	48	22.8	163	77.3	211	86.5	0.034
How much blood do we have in our body?	6	68.4	13	31.6	19	7.8	0.237
What is the composition of blood?	36	20.5	140	79.6	176	72.1	1.000
What are the existing blood groups?	28	23.9	89	76.1	117	48.0	0.209
What is the existing Rh factor?	25	18.8	108	81.2	133	54.5	0.525
How much blood can a person donate?	6	30.0	14	70.0	20	8.2	0.259
What is the minimum age to donate?	26	24.1	82	75.9	108	44.3	0.264
What is the minimum weight to donate blood?	16	17.0	78	83.0	94	38.5	0.330
What is the hemoglobin value that a man needs to have in order to donate blood?	25	21.4	92	78.6	117	48.0	0.754
What is the hemoglobin value a woman needs to have in order to donate blood?	19	24.7	58	75.3	77	31.6	0.307
How long before donating blood should alcoholic beverages be avoided?	35	20.7	134	79.3	169	69.3	1.000
How long does a person have to wait to donate blood after traveling abroad?	22	24.7	67	75.3	89	36.5	0.249
What recommendations should a person consider before donating blood?	5	17.2	24	82.8	29	11.9	0.808
How often can a man donate blood?	13	27.7	34	72.3	47	19.3	0.226
How often can a woman donate blood?	5	29.4	12	70.6	17	7.0	0.354
What are the most common adverse reactions to blood donation?	43	21.8	154	78.2	197	80.7	0.323
In what situations can women not donate blood?	32	18.8	138	81.2	170	69.7	0.388
What are the most at-risk groups that should not donate blood?	5	23.8	16	76.2	21	8.6	0.777
When might a blood transfusion be needed?	45	22.3	157	77.7	202	82.8	0.147
What infectious diseases can a person get from receiving a blood transfusion?	25	24.0	79	76.0	104	42.6	0.263

a Chi-square

## DISCUSSION

One of the main results of the study was the low score found in the knowledge assessment (mean number of correct answers of 8.90 [SD: 3.25] points out of a total of 21). It was evident that this score was significantly affected by the age, occupation, place of origin and educational level of the participants. Likewise, we didn’t find a relationship between knowledge and the history of donation.

Most of the questions associated with knowledge of the process and requirements for blood donation had a correct response frequency of less than 50%, showing a low level of knowledge. The mean overall score of the instrument was also low, which is consistent with several national and international studies ^12^ ^,^ ^16^ ^-^ ^18^. 

Literature suggests that a low level of knowledge can affect donor recruitment, but apparently this didn’t not occur in the study population. It is likely that, in this population, other factors are more closely related with the decision to donate, such as attitudes toward donation. Unfortunately, this study did not assess attitudes and did not explore motives for donation, which could have complemented our findings.

Our study found a lower frequency of donation history in women. This has been previously reported and could be explained by different situations specific to the sex, such as pregnancy or breastfeeding, as well as lower physiological hemoglobin levels, which could generate a worse perception about donation in women ^19^. Likewise, we found that knowledge related to the usefulness of blood was related to a greater history of donation. It is possible that people who understand the usefulness of blood also perceive its importance and the need for it in emergency situations, which would encourage them to donate.

The results of some studies suggest that belonging to some religions or religious sects affect the decision to donate blood in their members ^9^. In our study, most of the participants were Catholics, and a few belonged to other religions. However, we did identify a large number of religions, which prevents an adequate assessment of their impact on blood donation. Despite the above, our findings suggest that, in sects with a larger number of members, religion is not related to the history of donation, a fact that has been described in other similar settings ^20^.

The results of our study could possibly be extrapolated to insured workers with medium-low incomes in similar settings, i.e., cities located in jungle regions with a small number of inhabitants.

One of the limitations of the study was that the instrument was not validated with statistical tools, which could affect its representation of reality. Nevertheless, we do not believe that representation was significantly affected, since the items in the instrument corresponded to items in the existing national technical guide, which were corroborated by experts in the field. It is important to mention that some variables of interest such as religion or occupation are poorly represented, so that the data do not allow their adequate description and could have an undetected effect on the donation history or the level of knowledge.

The level of knowledge found in the population of Bagua Grande is low, but this would not be related to the history of donation. Interventions are needed to achieve a higher frequency of blood donation in order to meet the needs of the different populations. These interventions should provide knowledge to people, and should also take consider other aspects, since our study suggests that other factors, such as those social or cultural, have a strong influence on blood donation. We suggest conducting studies that evaluate the attitudes of similar populations about donation and the reasons why they donate, in order to achieve more effective interventions.
