# Supplementary material for: Knowledge about blood donation in patients of a hospital in Amazonas, Peru
Source: Rev Peru Med Exp Salud Publica. 2022 Jun 30;39(2):214–20. doi: 10.17843/rpmesp.2022.392.10829 (PMC11397686; doi:10.17843/rpmesp.2022.392.10829)
Supplement: Supplementary material. — Available in the electronic version of the RPMESP. [file rpmesp-39-02-10829-s001.docx]

**MATERIAL SUPLEMENTARIO**

**CUESTIONARIO DE CONOCIMIENTOS SOBRE DONACIÓN DE SANGRE**

Código: _____________________

1.Edad (en años): _________

2.Sexo

Femenino Masculino

3. Estado civil

Soltero Casado Viudo Divorciado

4. Ocupación

Empleado Jubilado Otros ____________________

5. Procedencia

Rural Urbano

6. Religión

Católico Otros

7. Grado de estudios

Sin estudios Primaria Secundaria Superior no universitaria

Superior Universitaria Estudios de posgrado

8. ¿Ha donado sangre alguna vez?

Sí No

Encierre en un círculo la letra que contenga la respuesta correcta.

1. ¿Dónde se forma la sangre?

a) en el músculo

b) en la médula ósea

c) en el corazón

d) en el estómago

e) no sabe

2. ¿Para qué sirve la sangre?

a) trasporta oxígeno a los tejidos del cuerpo

b) aumenta el crecimiento de los niños

c) mejora la función de los riñones

d) aumenta el peso de las personas

e) no sabe

3. ¿Cuánta sangre tenemos en nuestro cuerpo?

a) 4,5 a 6 litros

b) 4,5 a 5,5 litros

c) 5 a 6 litros

d) 5 a 7 litros

e) no sabe

4. ¿Cómo está compuesta la sangre?

a) glóbulos rojos, glóbulos blancos, plaquetas

b) glóbulos rojos, plasma, plaquetas

c) glóbulos rojos, plaquetas, eritrocitos

d) glóbulos rojos, glóbulos blancos, plasma

e) no sabe

5. ¿Cuáles son los grupos sanguíneos que existen?

a) grupo sanguíneo A y B

b) grupo sanguíneo AB

c) grupo sanguíneo O

d) grupo sanguíneo A, B, AB y O

e) no sabe

6. ¿Cuál es el factor Rh que existe?

a) factor Rh positivo

b) factor Rh negativo

c) factor Rh positivo y negativo

d) factor Rh nulo

e) no sabe

7. ¿Cuánta sangre puede donar una persona?

a) 430 mililitros

b) 450 mililitros

c) 500 mililitros

d) 550 mililitros

e) no sabe

8. ¿Cuál es la edad mínima para donar sangre?

a) 15 años

b) 18 años

c) 25 años

d) cualquier edad

e) no sabe

9. ¿Cuál es el peso mínimo para donar sangre?

a) 50 kilos

b) 55 kilos

c) 65 kilos

d) más de 80 kilos

e) no sabe

10. ¿Cuál es el valor de hemoglobina que un varón necesita tener para poder donar sangre?

1. Mayor o igual a 13,5 g/dL
2. Mayor o igual a 12,5 g/dL
3. Mayor o igual a 11,5 g/dL
4. Mayor o igual a 10,5 g/dL
5. no sabe

11. ¿Cuál es el valor de hemoglobina que una mujer necesita tener para poder donar sangre?

a) Mayor o igual a 13,5 g/dL

b) Mayor o igual a 12,5 g/dL

c) Mayor o igual a 11,5 g/dL

d) Mayor o igual a 10,5 g/dL

e) no sabe

12. ¿Cuánto tiempo antes de donar sangre no se debe ingerir bebidas alcohólicas?

a) 24 horas

b) 12 horas

c) 6 horas

d) 3 horas

e) no sabe

13. ¿Cuánto tiempo tiene que esperar una persona para donar sangre después de haber viajado al extranjero?

a) 1 año

b) 2 años

c) 3 años

d) 4 años

e) no sabe

14. ¿Qué recomendaciones sobre alimentación debe tener en cuenta una persona antes de donar sangre?

a) venir en ayunas

b) no beber líquidos

c) tomar leche

d) comer alimentos sin grasas

e) no sabe

15. ¿Cada cuánto tiempo puede donar sangre un varón?

a) Cada mes

b) Cada 3 meses

c) Cada 4 meses

d) Cada 6 meses

e) no sabe

16. ¿Cada cuánto tiempo puede donar sangre una mujer?

a) Cada mes

b) Cada 3 meses

c) Cada 4 meses

d) Cada 6 meses

e) no sabe

17. ¿Cuáles son las reacciones adversas más frecuentes a la donación de sangre?

a) sudoración

b) mareos

c) escalofríos

d) palpitaciones

e) no sabe

18. ¿En qué situaciones las mujeres no pueden donar sangre?

a) embarazo

b) después de dejar de lactar

c) menstruación

d) menopausia

e) no sabe

19. ¿Cuáles son los grupos de mayor riesgo que no deben donar sangre?

a) personas con cáncer

b) personas con diabetes

c) personas homosexuales

d) personas con presión alta

e) no sabe

20. ¿Cuándo se puede necesitar una transfusión de sangre?

a) en casos de cirugías

b) en casos de gripe

c) en casos de desmayos

d) en casos de ahogamiento

e) no sabe

21. ¿Qué enfermedades infecciosas puede contraer una persona al recibir una transfusión de sangre?

a) VIH, hepatitis, enfermedad de Chagas, sífilis

b) VIH, hepatitis, enfermedad de Chagas, tuberculosis

c) VIH, hepatitis, enfermedad de Chagas, diabetes

d) VIH, hepatitis, enfermedad de Chagas, tétanos

e) no sabe
