# Supplementary material for: Knowledge about blood donation in patients of a hospital in Amazonas, Peru
Source: Rev Peru Med Exp Salud Publica. 2022 Jun 30;39(2):214–20. doi: 10.17843/rpmesp.2022.392.10829 (PMC11397686; doi:10.17843/rpmesp.2022.392.10829)
Supplement: Translation

# Translation: Conocimientos sobre donación de sangre en pacientes de un hospital de Amazonas, Perú

**Authors:** Nelson Santisteban, Jorge Osada

**Affiliations:** 1 Hospital El Buen Samaritano, Seguro Social de Salud, Amazonas, Perú.; 2 Instituto de Evaluación de Tecnologías en Salud e Investigación, Seguro Social de Salud, Lima, Perú.

**Keywords:** Donantes de Sangre, Conocimiento, Servicio Ambulatorio en Hospital

## Abstract

El objetivo del estudio fue determinar el nivel de conocimientos sobre donación de sangre que tienen los pacientes externos que acuden a un hospital en Bagua Grande. Se realizó un estudio descriptivo transversal durante mayo a julio del 2019. Se elaboró y aplicó un cuestionario estructurado. Se captó a 244 personas que acudieron a consultorios externos mediante un muestreo sistemático. Los participantes obtuvieron una media de respuestas correctas de 8,90 (DE: 3,25) puntos de un total de 21. Se encontró que la edad y el grado de instrucción estaban correlacionados con el puntaje (rho = -0,21; p < 0,001 y rho = 0,38; p < 0,001, respectivamente). La procedencia y ocupación afectaron al puntaje (p < 0,001). Los conocimientos hallados en la población estudiada son bajos, pero esto no estaría relacionado con el antecedente de donación. Las intervenciones que se elaboren para mejorar la donación de sangre no solo se deben enfocar en brindar conocimientos, ya que otros factores estarían más relacionados con este resultado.

## INTRODUCCIÓN

La donación de sangre salva vidas a nivel mundial; sin embargo, esta práctica no es frecuente en América y tiene un bajo porcentaje. La Organización Panamericana de la Salud (OPS) y diversas organizaciones de salud de la región promueven la donación voluntaria de sangre. En el Perú, el Programa Nacional de Hemoterapia y Bancos de Sangre (PRONAHEBAS) ha realizado grandes esfuerzos para lograr un incremento en las donaciones voluntarias de sangre en la población, pero lamentablemente sus efectos no han sido importantes ^1^.

Las actitudes, las creencias y los conocimientos asociados con la donación de sangre pueden afectar la captación de donantes en la población. Así, esta se ve influenciada por factores como la educación y el contexto cultural^ (^ ^2^. La falta de conocimiento y los conceptos erróneos respecto a la donación pueden contribuir a la falta de iniciativa para la donación voluntaria^ (^ ^3^.

Diversos autores coinciden en que la población tiene conocimientos generales sobre la sangre, su transfusión y donación, pero tienen poco conocimiento sobre aspectos específicos del tema ^4^ ^-^ ^9^. Esta falta de información podría impedir que puedan tomar una decisión informada acerca de este proceso. Además, es común la presencia de tabúes y mitos sobre la donación de sangre los cuales influyen negativamente^ (^ ^10^. Entre dichas barreras encontramos el temor a la extracción, el contagio de enfermedades, comercialización de la sangre, religión, perdida de la fertilidad o desempeño sexual ^2^, la falta de necesidad de donadores, debilidad o anemia^ (^ ^10^.

Estudios realizados en Perú coinciden con lo reportado previamente, pero se enfocan predominantemente en poblaciones urbanas^ (^ ^11^ o relacionadas a las ciencias de la salud^ (^ ^12^ ^,^ ^13^, además la mayoría se publican como literatura gris. Si bien estos estudios brindan datos interesantes, sus resultados no se pueden extrapolar a la realidad nacional, debido a que esta se encuentra conformada por contextos culturales heterogéneos con diversidad de idiomas, dialectos y costumbres que podrían influir en los conocimientos, actitudes y experiencias de las personas sobre la donación de sangre. Bagua Grande es una ciudad con una gran actividad comercial ubicada en la selva peruana, que concentra personas de diversas zonas de la selva, lo que permite evaluar diversas costumbres y creencias regionales.

El objetivo de este trabajo fue determinar el nivel de conocimientos sobre la donación de sangre que tienen los pacientes que acuden al Hospital El Buen Samaritano de Bagua Grande. Asimismo, se exploró si este nivel de conocimientos se encontraba relacionado al antecedente de donación de sangre. Establecer dicho nivel de conocimientos permitirá realizar intervenciones específicas para mejorar la percepción de la donación de sangre y su frecuencia.

**MENSAJE CLAVE**

Motivación para realizar el estudio: la donación de sangre se da en una frecuencia baja, es necesario entender este problema para lograr un mejor acceso a esta necesidad.

Principales hallazgos: los conocimientos hallados en la población de Bagua Grande son bajos, estos no aparentan relación con el antecedente de donación.

Implicancias en salud pública: las intervenciones que se elaboren para mejorar la donación de sangre no solo se deben enfocar en brindar conocimientos, ya que otros factores estarían más relacionados con este resultado.

## EL ESTUDIO

Estudio descriptivo transversal ejecutado en un hospital de primer nivel de atención del seguro social peruano ubicado en la ciudad de Bagua Grande en la región Amazonas en Perú, durante el periodo de mayo a julio del 2019. Para establecer la muestra se consideró como población total a las personas que acudieron en el segundo trimestre del 2019 a los consultorios externos del hospital. Estas fueron 667 personas, según datos estadísticos suministrados por el mismo centro de salud. El cálculo muestral, considerando una proporción estimada del 50%, obtuvo una muestra de 244 personas. Asimismo, se aplicó un muestreo aleatorio sistemático, con un salto (k) de dos personas a todos los asistentes del área de consultorios externos del hospital que pasaban por el único acceso a dicha área.

Se elaboró un cuestionario estructurado (material suplementario) en base a la Guía Técnica para la Selección de Donante de Sangre Humana y Hemocomponentes, usada en el Perú ^14^ y los datos de estudios previos^ (^ ^15^. El cuestionario fue dividido en dos secciones; la primera incluyó características de los encuestados (edad, sexo, estado civil, ocupación, procedencia, religión, grado de estudios y antecedente de donación de sangre), y la segunda fue enfocada a medir conocimientos. Esta última sección se representó en 21 preguntas sobre la sangre, donación y sus procesos asociados. Todas las preguntas fueron de opciones múltiples cerradas, con excepción de la edad en años. Las preguntas de conocimientos solo tenían una respuesta correcta y cada respuesta positiva se consideró como un punto. El instrumento se validó por juicio de expertos quienes fueron profesionales especialistas en laboratorio, con experiencia y formación posgradual en el área. Se determinó el grado de acuerdo entre los expertos con el coeficiente Kappa de Fleiss. Se evaluó la confiabilidad de los ítems del instrumento mediante una prueba piloto a 60 personas que asistieron a otro hospital de la ciudad, aplicando el coeficiente de Kuder Richardson, que mostró un valor alto (0,77). Si bien existen instrumentos aplicados en otras realidades, se decidió crear un nuevo instrumento para ajustar los conocimientos a la guía técnica nacional. 

Se invitó a participar de la encuesta a personas mayores de 18 años que acudieron al área de consultorios externos del hospital y que tenían una cita programada en dichos consultorios o en servicios de ayuda diagnóstica. Mediante una preevaluación oral, se excluyó a quienes tuvieron enfermedades que impidan responder adecuadamente a las preguntas. Se invitó a la participación mediante un consentimiento informado. La encuesta fue llevada a cabo de lunes a sábado por los investigadores, en los horarios de trabajo del establecimiento de salud.

Se realizó un análisis descriptivo enfocado a presentar la frecuencia de conocimientos de forma global y de acuerdo con los datos sociodemográficos recolectados. Se exploró si las variables evaluadas estuvieron asociadas con el antecedente de donación mediante un análisis bivariado de acuerdo con las variables independientes elegidas y un análisis multivariado de Poisson robusto incluyendo a variables con un valor de p < 0,20 en el análisis bivariado. El análisis de datos se realizó utilizando el paquete estadístico Stata v15.

El estudio fue aprobado por el Comité de Investigación y Ética del Hospital Nacional Almanzor Aguinaga Asenjo de la Red Asistencial Lambayeque del Seguro Social de Salud (NIT: 4850-2019-684). La participación en el estudio fue voluntaria, y se presentó un consentimiento informado a todos los contactados. Los cuestionarios fueron almacenados separados de los formularios de consentimiento informado para garantizar el anonimato de los participantes durante el procesamiento de los datos.

## HALLAZGOS

Se evaluó al total de participantes planificados, es decir 244 individuos, los cuales tenían edades entre los 19 y 71 años, la mayoría fueron mujeres, contaban con grado de instrucción superior universitaria, poseían seguro obligatorio, eran empleados, residentes en zona urbana y profesaban la religión católica. Asimismo, la mayoría de los participantes negaron el antecedente de haber donado sangre. Se puede observar las características de los participantes en la Tabla 1.

**Tabla 1:** Media de puntaje de conocimientos de acuerdo con las características de los participantes

Características	n (244)	%	Media del puntaje	Valor de p ^a^
Edad ^b^	44,7	11,8	-0,2	<0,001 ^c^
Sexo				0,827
Femenino	159	65,2	8,9±3,1	
Masculino	85	34,8	8,8±3,5	
Estado civil				0,132 ^d^
Soltero	83	34,0	9,2±3,3	
Casado	81	33,2	8,2±3,4	
Conviviente	75	30,7	9,2±3,0	
Divorciado	3	1,2	11,7±2,3	
Viudo	2	0,8	10,0±2,8	
Tipo de seguro				<0,001 ^d^
Derechohabiente	60	24,6	7,5±3,3	
Obligatorio	166	68,0	9,6±2,8	
Pensionista	2	0,8	14,5±0,7	
Agrario	16	6,6	5,9±3,3	
Ocupación				<0,001^d^
Empleado	163	66,8	9,8±2,8	
Agricultor	18	7,4	5,9±3,7	
Ama de casa	49	20,1	6,8±2,9	
Estudiante	2	0,8	12,5±5,0	
Comerciante	1	0,4	6	
Independiente	8	3,3	8,3±2,4	
Jubilado	2	0,8	12,0±2,8	
Sin empleo	1	0,4	11	
Procedencia				<0,001
Urbano	188	77,1	9,3±3,0	
Rural	56	23,0	7,4±3,6	
Religión				0,698 ^d^
Católico	190	77,9	8,9±3,3	
Adventista	27	11,1	10,0±2,7	
Evangélica	1	0,4	4	
Israel de Jehová	1	0,4	9	
Mormón	1	0,4	9	
Movimiento Misionero Mundial (MMM)	2	0,8	8,0±1,4	
Nazareno	11	4,5	8,0±3,3	
Pentecostal	4	1,6	8,3±1,7	
Peregrino	3	1,2	7,7±4,2	
Testigo de Jehová	4	1,6	7,5±4,8	
Grado de Instrucción				<0,001^d^
Primaria	21	8,6	5,1±3,5	
Secundaria	59	24,1	7,3±2,7	
Superior no universitaria	74	30,3	10,1±2,8	
Superior universitaria	90	36,9	9,8±2,7	
Donación				0,096
No	194	79,5	8,7±3,3	
Sí	50	20,5	9,6±2,8	

a T de Student

b Media y desviación estándar

c Rho de Spearman

d Kruskal Wallis. Se usó una prueba no paramétrica debido al número pequeño de algunos grupos

La media de las respuestas correctas de los participantes fue de 8,90 (DE: 3,25) puntos, de un total posible de 21. Se puede observar el porcentaje de respuestas positivas para cada pregunta en la Tabla 2. Se exploró si las características de los participantes afectaron dicho puntaje, observándose que la edad se encontraba correlacionada de forma débil con el puntaje (rho = -0,21; p < 0,001), así como con el grado de instrucción (rho = 0,38; p < 0,001). Se notó un mayor nivel de conocimiento en las personas con seguro obligatorio y pensionistas (jubilados) que en derechohabientes (familiares del trabajador) y personas con seguro agrario, esto también se observó al comparar los empleados con las amas de casa y agricultores, y al comparar a personas con educación superior con las personas con educación primaria o secundaria. Asimismo, se observó una tendencia no significativa a tener un mayor conocimiento en las personas que tenían antecedente de donación (Tabla 1).

**Tabla 2:** Características de los participantes de acuerdo con el antecedente de donación

Características	Antecedente de donación				Valor de p
	Sí		No		
	n (50)	%	n (194)	%	
Edad ^a^	45,5	10,7	44,48	89,3	0,573^ b^
Sexo					0,007
Femenino	24	15,1	135	84,9	
Masculino	26	30,6	59	69,4	
Estado civil					0,116
Soltero	16	19,3	67	80,7	
Casado	11	13,6	70	86,4	
Conviviente	22	29,3	53	70,7	
Divorciado	1	33,3	2	66,7	
Viudo	0	0,0	2	100,0	
Tipo de seguro					0,622
Derechohabiente	9	15,0	51	85,0	
Obligatorio	38	22,9	128	77,1	
Pensionista	0	0,0	2	100,0	
Agrario	3	18,8	13	81,2	
Ocupación					0,135
Empleado	38	23,3	125	76,7	
Agricultor	4	22,2	14	77,8	
Ama de casa	4	8,2	45	91,8	
Estudiante	1	50,0	1	50,0	
Comerciante	0	0,0	1	100,0	
Independiente	3	37,5	5	62,5	
Jubilado	0	0,0	2	100,0	
Sin empleo	0	0,0	1	100,0	
Procedencia					0,707
Urbano	40	21,3	148	78,7	
Rural	10	17,9	46	82,1	
Religión					0,249
Católico	39	20,5	151	79,5	
Adventista	5	18,5	22	81,5	
Evangélica	0	0,0	1	100,0	
Israel de Jehová	1	100,0	0	0,0	
Mormón	0	0,0	1	100,0	
Movimiento Misionero Mundial (MMM)	1	50,0	1	50,0	
Nazareno	1	9,1	10	90,9	
Pentecostal	1	25,0	3	75,0	
Peregrino	2	66,7	1	33,3	
Testigo de Jehová	0	0,0	4	100,0	
Grado de Instrucción					0,700
Primaria	3	14,3	18	85,7	
Secundaria	10	17,0	49	83,1	
Superior no universitaria	18	24,3	56	75,7	
Superior universitaria	19	21,1	71	78,9	

a Media y desviación estándar

b T de Studentb T de Student

Se exploró si las características de los participantes estaban relacionadas con el antecedente de donación de sangre. El sexo estuvo relacionado significativamente con dicho evento, pues se encontró un mayor antecedente de donación en hombres. No se observó asociación con el resto de las variables (Tabla 2). Al explorar la relación entre la frecuencia de respuestas correctas a las preguntas de conocimiento y el antecedente de donación de sangre, solo se observó relación con la pregunta 2 (¿para qué sirve la sangre?) (Tabla 3). Se realizó un análisis multivariado, pero no se observaron diferencias en relación al bivariado. Asimismo, los subgrupos para algunas variables fueron muy pequeños como para hacer un análisis aceptable (datos no mostrados).

**Tabla 3:** Conocimientos de los participantes de acuerdo con el antecedente de donación

Conocimientos (respuestas correctas)	Antecedente de donación						Valor de p^ a^
	Sí		No		Total		
	n (50)	%	n (194)	%	n (244)	%	
¿Dónde se forma la sangre?	14	25,9	40	74,1	54	22,1	0,258
¿Para qué sirve la sangre?	48	22,8	163	77,3	211	86,5	0,034
¿Cuánta sangre tenemos en nuestro cuerpo?	6	68,4	13	31,6	19	7,8	0,237
¿Cómo está compuesta la sangre?	36	20,5	140	79,6	176	72,1	1,000
¿Cuáles son los grupos sanguíneos que existen?	28	23,9	89	76,1	117	48,0	0,209
¿Cuál es el factor Rh que existe?	25	18,8	108	81,2	133	54,5	0,525
¿Cuánta sangre puede donar una persona?	6	30,0	14	70,0	20	8,2	0,259
¿Cuál es la edad mínima para donar?	26	24,1	82	75,9	108	44,3	0,264
¿Cuál es el peso mínimo para donar sangre?	16	17,0	78	83,0	94	38,5	0,330
¿Cuál es el valor de hemoglobina que un varón necesita tener para poder donar sangre?	25	21,4	92	78,6	117	48,0	0,754
¿Cuál es el valor de hemoglobina que una mujer necesita tener para poder donar sangre?	19	24,7	58	75,3	77	31,6	0,307
¿Cuánto tiempo antes de donar sangre no se debe ingerir bebidas alcohólicas?	35	20,7	134	79,3	169	69,3	1,000
¿Cuánto tiempo tiene que esperar una persona para donar sangre después de haber viajado al extranjero?	22	24,7	67	75,3	89	36,5	0,249
¿Qué recomendaciones debe tener en cuenta una persona antes de donar sangre?	5	17,2	24	82,8	29	11,9	0,808
¿Cada cuánto tiempo puede donar sangre un varón?	13	27,7	34	72,3	47	19,3	0,226
¿Cada cuánto tiempo puede donar sangre una mujer?	5	29,4	12	70,6	17	7,0	0,354
¿Cuáles son las reacciones adversas más frecuentes a la donación de sangre?	43	21,8	154	78,2	197	80,7	0,323
¿En qué situaciones las mujeres no pueden donar sangre?	32	18,8	138	81,2	170	69,7	0,388
¿Cuáles son los grupos de mayor riesgo que no deben donar sangre?	5	23,8	16	76,2	21	8,6	0,777
¿Cuándo se puede necesitar una transfusión de sangre?	45	22,3	157	77,7	202	82,8	0,147
¿Qué enfermedades infecciosas puede contraer una persona al recibir una transfusión de sangre?	25	24,0	79	76,0	104	42,6	0,263

a Chi cuadrado

## DISCUSIÓN

Entre los principales hallazgos del estudio se observa un bajo puntaje en la evaluación de conocimientos (media de respuestas correctas de 8,90 [DE: 3,25] puntos de un total de 21). Se evidenció que este puntaje estaba afectado significativamente por la edad, ocupación, origen y grado de instrucción de los participantes. Asimismo, al explorar la relación entre los conocimientos evaluados y el antecedente de donación se observó que estos no estaban relacionados.

La mayoría de las preguntas asociadas al conocimiento del proceso y requerimientos para la donación de sangre tienen una frecuencia de respuesta correcta de menos del 50%, lo que indica un bajo conocimiento. La media del puntaje global del instrumento también es baja, lo cual es consistente con varios estudios nacionales e internacionales ^12^ ^,^ ^16^ ^-^ ^18^. 

La literatura sugiere que un bajo nivel de conocimiento puede afectar a la captación de donantes en la población, pero aparentemente este hecho no se está dando en la población de estudio. Es probable que, en esta población, otros factores se vean más involucrados en la decisión de donación como las actitudes hacia este hecho. Lamentablemente el presente estudio no consideró la evaluación de actitudes y tampoco exploró los motivos de la donación, que podrían complementar nuestros hallazgos.

En este estudio se evidencio una menor frecuencia de antecedente de donación en mujeres. Esto ya se ha reportado previamente y se podría explicar por diversas situaciones propias del sexo como el embarazo o la lactancia, así como niveles fisiológicos menores de hemoglobina, lo que generaría una peor percepción de la donación en este sexo ^19^. Asimismo, se observó que el conocimiento de la utilidad de la sangre estaba relacionado con un mayor antecedente de donación. Posiblemente las personas que entiendan la utilidad de la sangre perciban también su importancia y la necesidad de esta en situaciones de emergencia lo que los incentivaría a la donación.

Los resultados de algunos estudios sugieren que pertenecer a algunas religiones o sectas religiosas afectan la decisión de donación de sangre en sus miembros ^9^. En nuestro estudio no se han observado diferentes religiones en la población, la mayoría de los participantes fueron católicos. Se han evidenciado una gran cantidad de religiones, lo cual impide hacer una evaluación adecuada de su impacto sobre la donación de sangre. A pesar de lo mencionado, nuestros hallazgos sugieren que, en las sectas con un mayor número de miembros, la religión no está relacionada con el antecedente de donación, hecho que se ha descrito en otras realidades similares ^20^.

Los resultados del estudio posiblemente sean extrapolables a trabajadores asegurados con ingresos medio-bajos, atendidos en realidades similares, es decir, ciudades ubicadas en regiones selváticas con un número pequeño de habitantes. 

Entre las limitaciones del estudio se debe mencionar que el instrumento utilizado no se validó con herramientas estadísticas, lo que podría afectar su representación de la realidad. A pesar de ello no creemos que se haya afectado de forma importante dicha representación ya que los ítems del instrumento correspondían a puntos de la guía técnica nacional existente, que fueron corroborados por expertos del área. Es pertinente mencionar que algunas variables de interés como religión u ocupación están poco representadas, por lo que estos datos no permiten su descripción adecuada y podrían tener un efecto no detectado sobre el antecedente de donación o los conocimientos estudiados.

Los conocimientos hallados en la población de Bagua Grande son bajos, pero esto no estaría relacionado con el antecedente de donación. Es necesario realizar intervenciones que permitan lograr una frecuencia alta de donación de sangre para cumplir con las necesidades de cada realidad. Dichas intervenciones deben brindar conocimientos a las personas, además deben tener en cuenta otros factores, puesto que nuestro estudio sugiere que otros factores, como los sociales o culturales, influyen de gran manera en la donación de sangre. Sugerimos realizar estudios que evalúen las actitudes de poblaciones similares sobre la donación y los motivos por los cuales se realizan, a fin de lograr intervenciones más efectivas.
